# Whole-Genome Sequence and Fosfomycin Resistance of *Bacillus* sp. Strain G3(2015) Isolated from Seawater off the Coast of Malaysia

**DOI:** 10.1128/genomeA.00067-17

**Published:** 2017-03-30

**Authors:** Xin-Yue Chan, Jian-Woon Chen, Tan-Guan-Sheng Adrian, Kar-Wai Hong, Chien-Yi Chang, Wai-Fong Yin, Kok-Gan Chan

**Affiliations:** a Division of Genetics and Molecular Biology, Institute of Biological Sciences, Faculty of Science, University of Malaya, Kuala Lumpur, Malaysia; b UM Omics Centre, University of Malaya, Kuala Lumpur, Malaysia; c Institute of Life and Earth Sciences, Heriot Watt University, Edinburgh, United Kingdom; d Guangzhou Institute of Pediatrics, Guangzhou Women and Children’s Medical Center, Guangzhou Medical University, Guangzhou, Guangdong, China

## Abstract

*Bacillus* sp. is a Gram-positive bacterium that is commonly found in seawater. In this study, the genome of marine *Bacillus* sp. strain G3(2015) was sequenced using MiSeq. The fosfomycin resistant gene *fosB* was identified upon bacterial genome annotation.

## GENOME ANNOUNCEMENT

The high adaptability of *Bacillus* spp. enables their survival in a wide range of environments, including environments with extreme temperature, pressure, salinity, and pH (1). The production of diverse classes of antibiotics by *Bacillus* spp. has enhanced their ability to compete for survival in diverse environments (2, 3). In contrast to its extensively studied terrestrial counterparts, less research has been conducted on marine *Bacillus* spp. In this study, we present the whole genome of marine *Bacillus* sp. strain G3(2015) and its antibiotic resistance gene.

*Bacillus* sp. G3(2015) was isolated from subsurface seawater collected from the coast of Georgetown, Malaysia. Genomic DNA was extracted from *Bacillus* sp. G3(2015) using the MasterPure Gram-positive DNA purification kit (Epicenter, USA). The extracted DNA was quantified using a Qubit 2.0 Fluorometer (Thermo Fisher Scientific, USA) while the quality of the DNA was verified using a NanoDrop spectrophotometer (Thermo Fisher Scientific, USA) (4–6). Sequencing library preparation was prepared using a Nextera DNA library preparation kit (Illumina, USA). Whole-genome sequencing was performed on the MiSeq sequencer (Illumina, USA) using MiSeq reagent kit v2. Paired-end reads generated from MiSeq were trimmed and *de novo* assembled using CLC Genomic Workbench (v8). Gene prediction was carried out using Prodigal (v2.6.1) (7). The bacterial genome was then annotated using the NCBI Prokaryotic Genome Annotation Pipeline (v3) and Rapid Annotations using Subsystems Technology (RAST) (v2) (8, 9). The predicted gene sequences were also aligned using BLAST against the Antibiotic Resistance Genes Database (ARDB) to determine the presence of antibiotic resistance genes (10).

The bacterial genome was assembled into 120 contigs with an average coverage of 75-fold. The *N*_50_ of the contigs is 83,170 bp. The estimated genome size is 5.4 Mbp with 34.9% G+C content. A total of 5,565 coding sequences (CDS), eight rRNAs, 96 tRNAs, and 99 pseudogenes were identified from the draft genome.

Fosfomycin resistance gene (*fosB*) (accession number WP_000943769) was detected in contig 76 of the genome (NZ_LKID01000076.1) of *Bacillus* strain G3(2015) by both RAST and BLAST against ARDB. Fosfomycin is an antibiotic functionally effective against Gram-positive and Gram-negative bacteria by irreversibly inhibiting bacterial cell wall biosynthesis (11, 12). It is mainly prescribed for the treatment of uncomplicated urinary tract infections and gastrointestinal infections (12). The *fosB* gene synthesizes thiol-S-transferase (FosB) which catalyzes the addition of bacillithiol to the fosfomycin epoxide ring leading to the inhibition of fosfomycin (13). Studies have shown that the *fosB* gene is transferable across genera via conjugation (14, 15). The high bacterial diversity in tropical seawater could increase the rate of emergence of fosfomycin resistant strains from different genera. Therefore, this study on fosfomycin resistant *Bacillus* sp. strain G3(2015) that was isolated from seawater is important, especially in the context of public health.

### Accession number(s).

This whole-genome shotgun project has been deposited in DDBJ/EMBL/GenBank under the accession no. LKID00000000. The version described in this paper is the first version, LKID01000000.
